# Is *LMNB1* a Susceptibility Gene for Neural Tube Defects in Humans?

**DOI:** 10.1002/bdra.23141

**Published:** 2013-06-03

**Authors:** Alexis Robinson, Darren Partridge, Ashraf Malhas, Sandra CP De Castro, Peter Gustavsson, Dominic N Thompson, David J Vaux, Andrew J Copp, Philip Stanier, Alexander G Bassuk, Nicholas DE Greene

**Affiliations:** 1 Neural Development Unit and Newlife Birth Defects Research Centre, Institute of Child Health, University College LondonUnited Kingdom; 2 Sir William Dunn School of Pathology, University of OxfordUnited Kingdom; 3 Department of Molecular Medicine and Surgery, Karolinska InstitutetStockholm, Sweden; 4 Department of Neurosurgery, Great Ormond Street Hospital for Children NHS TrustLondon, United Kingdom; 5 Department of Pediatrics, University of IowaIowa City, Iowa

**Keywords:** anencephaly, lamin B1, neural tube defects, nuclear lamina, spina bifida

## Abstract

**BACKGROUND:**

Lamins are intermediate filament proteins that form a major component of the nuclear lamina, a protein complex at the surface of the inner nuclear membrane. Numerous clinically diverse conditions, termed laminopathies, have been found to result from mutation of *LMNA*. In contrast, coding or loss of function mutations of *LMNB1*, encoding lamin B1, have not been identified in human disease. In mice, polymorphism in *Lmnb1* has been shown to modify risk of neural tube defects (NTDs), malformations of the central nervous system that result from incomplete closure of the neural folds.

**METHODS:**

Mutation analysis by DNA sequencing was performed on all exons of *LMNB1* in 239 samples from patients with NTDs from the United Kingdom, Sweden, and United States. Possible functional effects of missense variants were analyzed by bioinformatics prediction and fluorescence in photobleaching.

**RESULTS:**

In NTD patients, we identified two unique missense variants that were predicted to disrupt protein structure/function and represent putative contributory mutations. Fluorescence loss in photobleaching analysis showed that the A436T variant compromised stability of lamin B1 interaction within the lamina.

**CONCLUSION:**

The genetic basis of human NTDs appears highly heterogenous with possible involvement of multiple predisposing genes. We hypothesize that rare variants of *LMNB1* may contribute to susceptibility to NTDs. *Birth Defects Research (Part A) 97:398–402, 2013*. © 2013 Wiley Periodicals, Inc.

## INTRODUCTION

Lamins are intermediate filament proteins that are key components of the nuclear lamina, a meshwork of lamin polymers and lamin-binding proteins that underlies the inner nuclear membrane (Capell and Collins, 2006; Gruenbaum et al., 2005; Worman et al., 2009). *LMNA* encodes lamin A and C (A-type lamins), while the B-type lamins are encoded by *LMNB1* (lamin B1) and *LMNB2* (lamins B2 and B3).

Genomic duplication of *LMNB1* causes adult-onset autosomal dominant leukodystrophy, a progressive demyelinating disorder (Padiath et al., 2006; Schuster et al., 2011). However, coding or loss-of-function mutations of *LMNB1* have not yet been identified in human disease. This is in marked contrast to *LMNA*, mutation of which results in several clinically distinct diseases, termed laminopathies. These include progeria syndromes (e.g., Hutchinson-Gilford progeria syndrome and Atypical Werner syndrome), muscular dystrophy disorders (e.g., Emery-Dreyfus muscular dystrophy), lipodystrophies, and Charcot-Marie-Tooth disease type 2B1, a peripheral neuropathy (Capell and Collins, 2006; Worman et al., 2010). Mutations in *LMNB2* may also contribute to acquired partial lipodystrophy (Hegele et al., 2006).

A role for B-type lamins in nervous system development was indicated by the finding of neuronal migration defects and consequent cortical abnormalities in *Lmnb1* and *Lmnb2* knockout mice (Coffinier et al., 2010; Coffinier et al., 2011), while forebrain-specific *Lmnb1/Lmnb2* double mutants exhibit cortical atrophy. A possible requirement for lamin B1 function in early development of the central nervous system was recently highlighted by the identification of *Lmnb1* as a possible modifier gene for neural tube defects (NTDs) in mice (De Castro et al., 2012). A polymorphic variant of *Lmnb1* was found to be present on the genetic background of the *curly tail* strain, in which embryos develop partially penetrant spinal and cranial NTDs due to incomplete closure of the neural tube. This variant (Deletion 18: 56909394) contains a series of eight instead of nine glutamic acid residues in the C-terminal domain of the protein, leading to increased mobility in the lamina. There was a corresponding increase in numbers of dysmorphic nuclei and premature senescence in fibroblasts expressing the variant lamin B1 (De Castro et al., 2012), reminiscent of the cellular phenotype of *Lmnb1* null fibroblasts (Vergnes et al., 2004). The principal genetic cause of NTDs in the *curly tail* strain is homozygosity for a hypomorphic allele of *grainyhead-like-3* (*Grhl3*) (Gustavsson et al., 2007). However, breeding the wild-type *Lmnb1* onto the *curly tail* strain background resulted in a threefold reduction in the frequency of spina bifida and exencephaly (De Castro et al., 2012).

In the current study, we investigated a possible role for *LMNB1* mutation in human NTDs, which are among the most common birth defects, affecting around 1 per 1000 pregnancies worldwide, with higher rates in some regions. Elucidation of the causes of NTDs is problematic owing to their complex, multifactorial etiology and largely sporadic nature (Bassuk and Kibar, 2009; Greene et al., 2009). The defining feature of NTDs, such as spina bifida and anencephaly, is the failure of closure of the neural tube during embryonic development (Copp and Greene, 2010). This process is dependent on coordinated shaping, bending, and fusion of the neural folds (Greene and Copp, 2009). The sensitivity of these events to genetic disruption is exemplified by the fact that individual mutation of more than 200 different genes has been found to result in NTDs in mice (Copp et al., 2003; Harris and Juriloff, 2007; Harris and Juriloff, 2010).

Several lines of evidence indicate that there is a genetic component in human NTDs, the clearest indication being the progressive increase in recurrence risk following affected pregnancies (Harris and Juriloff, 2007). Susceptibility to NTDs is also influenced by environmental factors. These include maternal diabetes or use of anti-epileptic medication, which are known to exacerbate risk, or maternal use of folic acid supplements, which is protective. Identification of these risk factors provided impetus for extensive analysis of genes related to glucose and folate metabolism in the causation of NTDs. Associations have been reported between genes involved with glucose metabolism and susceptibility to spina bifida (Davidson et al., 2008; Lupo et al., 2012). Several genes related to folate metabolism have also shown associations with risk of NTDs (reviewed by Boyles et al., 2005; Blom et al., 2006; Greene et al., 2009; Shaw et al., 2009). In addition to association studies, sequencing analysis has been performed on numerous candidate genes, implicated either by known environmental risk factors in humans or by the presence of NTDs in mouse models. For example, loss of function mutations in components of the glycine cleavage system, a constituent of mitochondrial folate metabolism, have been identified in NTD patients and disruption of the glycine cleavage system through knockout of *Amt* also causes NTDs in mice (Narisawa et al., 2012). Loss of function mutations in genes encoding components of the noncanonical Wnt signaling pathway (planar cell polarity pathway) causes craniorachischisis in mice, and predisposes to spina bifida or anencephaly in some digenic models (reviewed in Greene et al., 2009; Juriloff and Harris, 2012). Analysis of planar cell polarity genes in humans has revealed mutations in *SCRIB* and *CELSR1* in craniorachischisis cases (Robinson et al., 2012), while mutations in *VANGL1*, *VANGL2*, *CELSR1*, and *FZD6* have also been identified in spina bifida and anencephaly cases (Kibar et al., 2007; Kibar et al., 2009; Lei et al., 2010; Allache et al., 2012; De Marco et al., 2012). To date, no individual gene has been found to be mutated in a more than a small percentage of NTD patients, supporting the idea of wide genetic heterogeneity. Nevertheless, these studies encourage the view that mouse models provide insight into the genetic etiology of human NTDs.

## MATERIALS AND METHODS

### Patient Cohorts and Sequencing

Mutation analysis by DNA sequencing was performed on all exons of *LMNB1* (accession no. L37737.2; HGNC: 6637). Cases comprised a total of 239 samples from patients with NTDs collected, with ethical permission, in the United Kingdom (n = 65; Newcastle upon Tyne Hospital, Queen Charlotte's and Chelsea Hospital, Great Ormond Street Hospital for Sick Children), Sweden (n = 76; Karolinska University Hospital), and the United States (n = 98; Northwestern University, Children's Memorial Hospital, Illinois and Greenwood Genetics Center, South Carolina). In the majority of cases, the phenotype was open spina bifida (myelomeningocele; n = 233), with a few cases of anencephaly (n = 3) and encephalocele (n = 3). Exons containing missense variants were also sequenced in a cohort of 192 well-characterized U.K. controls (Apostolidou et al., 2007) and a cohort of 184 Swedish controls.

### Sequencing

Genomic DNA fragments spanning exons and exon–intron boundaries of *LMNB1* were amplified by PCR (11 exons, 1758-bp open reading frame). Purified PCR products were sequenced using big dye terminator chemistry (Applied Biosystems) and analyzed on a 3100 Genetic Analyzer (ABI). Sequence reads derived from both strands were assembled, aligned, and analyzed for nucleotide differences using Sequencher v4.8 (GeneCodes). Unique variants were checked by repeat PCR and re-sequencing. Variants were assessed for frequency in the Exome Variant Server (National Heart, Lung, and Blood Institute GO Exome Sequencing Project [ESP]; Seattle, WA; http://evs.gs.washington.edu/EVS/; data release ESP6500, November 2012).

### Bioinformatic Analysis

The predicted effect of amino acid variants on protein structure/function was assessed using PolyPhen-2 (v2.2.2r398) and potential effects on splicing were predicted using Alamut software (v2.0). Lamin B1 protein sequences from multiple species were aligned using MultAlin software (Corpet, 1988). The aligned sequence was used to model the secondary structure and relative accessibility of residues in the globular tail domain (PDB ID 3UMN: residues 428–550) using tools in ESPript v2.2 (Gouet et al., 1999) and the protein data bank (http://www.pdb.org). Prediction of the effect of amino acid variants on local phosphorylation sites was performed using GPS2.1.

### Fluorescence Loss in Photobleaching

Constructs were generated in the pcDNA3.1 vector by standard cloning methods, to express fusion proteins composed of a nuclear localization signal, yellow fluorescent protein, and 200 amino acids of the lamin B1 C-terminal region. Missense variants were introduced by site-directed mutagenesis. Plasmids were transfected into HeLa cells and fluorescence loss in photobleaching was performed as described previously (Malhas et al., 2009). In brief, a region of interest was photobleached at full laser power while scanning at 4% laser power elsewhere. For quantitative analysis, background intensity was subtracted, and intensities of a specific region of interest outside the photobleached area were measured over time and normalized using intensities of an region of interest in a transfected but nonbleached cell.

## RESULTS

**Figure 1:**
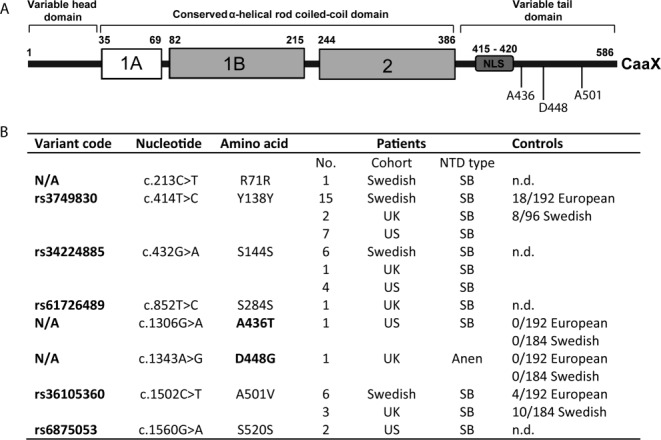
*LMNB1* sequence variants identified in neural tube defect (NTD) patients. (**A**) Schematic diagram of lamin B1 protein structure shows the head and tail domains that mediate polymer assembly and the central rod, comprising coiled-coli domains (*1A*, *1B*, and *2*). A nuclear localization signal (*NLS*) is present in the tail domain. The positions of missense variants identified among patients are indicated. (**B**) Sequencing of *LMNB1* (NM_005573.2 used as reference sequence) in 239 NTD patients revealed eight different variants (five synonymous and three nonsynonymous) present in heterozygous form in a total of 48 individuals (2 patients carried two heterozygous variants). None of the variants were predicted to affect splicing. Two variants were present in NTDs but not in 376 controls sequenced in this study or in the Exome Variant Server database. Frequency of previously reported variants in the Exome Variant Server database were rs3749830, C=700/T=12,306; rs34224885, A=355/G=12,651; rs61726489, C=55/T=12,951; rs36105360, T=252/C=12,754; rs6875053, A=527/G=12,479.

Exon sequencing of *LMNB1* revealed several variants in NTD patients (Fig. 1), each of which was present in heterozygous form. Variants included five synonymous and three nonsynonymous alterations. Four of the synonymous variants corresponded to known single nucleotide polymorphisms and one, c.213C>T (R71R), had not previously been reported in public variant databases. Three of the synonymous variants were present in two or more patients (24/239 in the case of rs3749830) and the other two, including the previously unreported variant, were only detected in single individuals.

Three nonsynonymous (missense) alterations were detected. Among these A501V corresponds to a previously reported single nucleotide polymorphism, rs36105360, and was detected in nine patients. In contrast, the two other variants A436T and D448G were each detected in only one individual, one with spina bifida and one with anencephaly. Neither variant was present in 376 controls (752 chromosomes), the single nucleotide polymorphisms database (dbSNP), or in the National Heart, Lung, and Blood Institute Exome Sequencing Project/Exome Variant Server Database. Alignment of protein sequences revealed that the alanine (A436) and aspartic acid (D448) residues found to vary in NTD patients are highly conserved (in human, chimpanzee, macaque, mouse, rat, dog, chick, frog, and zebra fish). Bioinformatics analysis indicated that the polymorphic A501V change is predicted to be a “benign” variant, whereas the A436T and D448G variants, which were unique to NTDs, are both predicted to be “probably damaging.” Both variants are located in the globular tail domain of the lamin B1 protein, residues 428 to 550 (Fig. 1A). In silico modeling of the secondary structure of the tail domain suggests that A436 is a surface residue and D448 is partially buried (data not shown). A436T lies in an area rich in serine/threonine potential phosphorylation sites. Bioinformatics prediction of the effect of the A436T variant on local phosphorylation sites (using GPS2.1) indicates possible effects on the phosphorylation of S431, S433, and S437. D448 lies 10 amino acid residues from the closest predicted phosphorylation sites (T439 and T459), and the D448G variant is not predicted to affect their modification.

**Figure 2:**
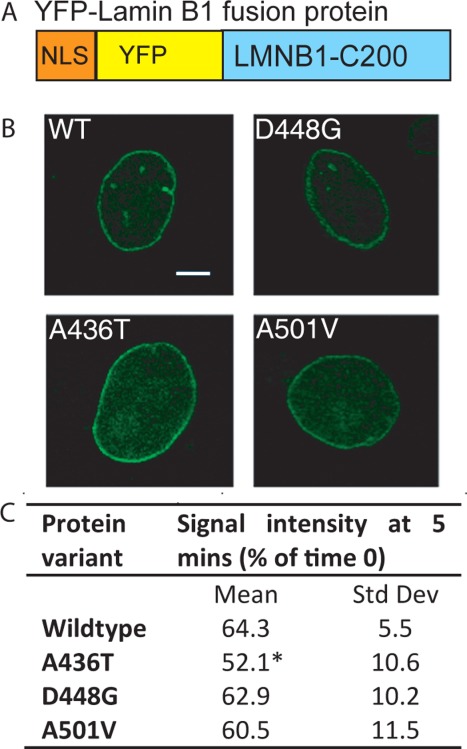
Functional analysis of lamin B1 missense variants by fluorescence loss in photobleaching. (**A**) HeLa cells were transfected with constructs expressing fusion proteins comprising a nuclear localization signal, yellow fluorescent protein (*YFP*), and the C-terminal 200 amino acids of lamin B1, which corresponds to the tail domain. (**B**) The wild-type and mutant fusion proteins all localize to the nuclear lamina. (**C**) FLIP analysis showed similar loss of signal intensity in the lamina of cells expressing fusion proteins with the wild-type lamin B1 sequence and the D448G or A501V variants. The A436T variant showed a significantly greater decline in signal intensity (*P* < 0.01; *t*-test), indicating greater mobility in the nuclear lamina. Scale bar = 5 μm. [Color figure can be viewed in the online issue, which is available at wileyonlinelibrary.com.]

To further examine the possible functional effects of the missense variants identified in NTD patients, we used fluorescence loss in photobleaching to analyze their effect on lamin B1 stability in the nuclear lamina. This approach relies on photobleaching of fluorescently tagged proteins in one area of the nuclear lamina and monitoring of loss of fluorescent intensity in an adjacent unbleached area as proteins relocate within the lamina. Fusion proteins comprising a nuclear localization sequence, yellow fluorescent protein, and the 200 C-terminal residues of lamin B1 (Fig. 2A) were expressed in HeLa cells, as previously performed (Malhas et al., 2007). All of the variant proteins localized to the nuclear envelope as observed for the wild-type protein (Fig. 2B). However, following photobleaching, there was a more rapid decline in fluorescence intensity in the unbleached area of membrane in cells expressing the A436T variant compared with wild-type (Fig. 2C). This behavior is indicative of increased mobility (Malhas et al., 2007) and, hence, decreased stability of interaction of the A436T variant within the nuclear envelope. Neither D448G nor A501V showed a significant difference in the decline in signal intensity to the wild-type protein (Fig. 2C).

## DISCUSSION

Pinpointing the genetic basis of NTDs is particularly challenging owing to their multigenic inheritance and potential influence of environmental factors. Thus, unlike many NTD mouse models in which defects arise in homozygous null embryos, NTDs in humans rarely show Mendelian inheritance. It appears likely that a defect in a given individual may arise from summation of two or more factors that are individually insufficient to cause NTDs. Where putative contributory mutations have been identified, for example in genes of the planar cell polarity pathway or glycine cleavage system, they are present in heterozygous form and, where evaluated, have usually been found to be inherited from an unaffected parent (reviewed by Narisawa et al., 2012). This suggests that these NTD patients also carry variant alleles in other genes, consistent with the multigenic threshold model of inheritance of NTDs (Harris and Juriloff, 2007). We propose that *LMNB1* may represent one such “susceptibility” gene in which rare variants contribute to NTD predisposition in some individuals.

Whereas only a small proportion of NTD patients exhibited putative mutations in *LMNB1*, two missense variants were unique to NTD patients and both were predicted to be damaging. We could not determine whether the mutations arose de novo, as parental samples were not available. In the case of A436T, we also found a direct effect of the variant, compromising the stability of the protein's interaction in the nuclear lamina. Such an effect was also observed for the variant found to increase susceptibility to NTDs in mice (De Castro et al., 2012). Lamins have multiple functions in the nuclear envelope and influence a variety of cellular properties. There is a structural role of lamins in assembly and maintenance of the nuclear envelope, nuclear shape, and anchoring of nuclear pore complexes (Hutchison, 2002; Dechat et al., 2008). Lamin B1 has additional functions in DNA synthesis and transcriptional regulation, mediated through interactions with both chromatin and transcription factors (Malhas et al., 2007; Malhas et al., 2010; Mekhail and Moazed, 2010). Dysregulation of cellular proliferation and/or transcriptional regulation both represent potential mechanisms by which impairment of lamin B1 function could impact on neural tube closure.

In summary, we hypothesize that mutations in *LMNB1* may contribute to susceptibility to NTDs in a subset of patients. Although the majority of cases analyzed in the current study were spina bifida, we note that one of the mutations was present in a patient with anencephaly (cranial NTDs), suggesting that further analysis of *LMNB1* may be particularly worthwhile in this group of NTDs. Identification of susceptibility genes for NTDs will assist in understanding the genetic basis of NTDs in affected families and may inform development of possible preventive approaches.
